# Intensified research on tuberculosis in the Western Pacific Region: a bibliometric analysis, 2000–2019

**DOI:** 10.5365/wpsar.2020.11.3.003

**Published:** 2020-12-28

**Authors:** Fukushi Morishita, Takuya Yamanaka, Tauhid Islam

**Affiliations:** a End TB and Leprosy Unit, World Health Organization Regional Office for the Western Pacific, Manila, Philippines.; b Department of Global Health and Development, London School of Hygiene and Tropical Medicine, London, United Kingdom of Great Britain and Northern Ireland.

## Abstract

“Intensified TB research and innovation” is one of the three pillars of the End TB Strategy. To assess achievements and gaps in tuberculosis (TB) research productivity in countries and areas of the Western Pacific Region quantitatively, a bibliometric analysis was carried out by examining trends in the numbers of publications on TB indexed in PubMed between 2000 and 2019 and by comparing them with trends in publications on other selected major infectious diseases for the same period. The number of publications on TB in the Region increased by 3.2 times during the period, from 534 in 2000–2004 to 1714 in 2015–2019, as compared with 2.9 times each for HIV, hepatitis and malaria. The number increased by 46% in 2005–2009, 79% in 2010–2014 and 23% in 2015–2019, as compared with each previous 5-year period. The average annual growth rate between 2000 and 2018 was 8.8%. China accounted for 34.8% of the total number of publications on TB in the Region. Increases in TB research were observed in most countries and areas in the Region, particularly in those with a high TB burden. The number of publications on TB remained low, however, in the Lao People's Democratic Republic, Mongolia and Pacific island countries. Countries are encouraged to implement the set of actions proposed in the Global Strategy for TB Research and Innovation to accelerate progress towards ending TB.

Tuberculosis (TB) remains a major public health issue globally. In 2018, worldwide, an estimated 10 million people contracted TB and 1.5 million died from the disease. (*1*) Since 2015, the WHO End TB Strategy has guided national TB responses by providing principles and essential programme components in three fundamental pillars. (*2*) The Strategy set ambitious targets for ending TB: reducing the incidence by 90% and deaths by 95% in 2035, as compared with 2015, and eliminating catastrophic costs for TB-affected households. (*2*) To reach these targets, new tools and strategies must be developed and introduced, with universal access to and better use of existing technologies. (*3*) The third pillar of the Strategy, “intensified research and innovation,” thus promotes intensification of research on TB at all levels and empowerment of a strong, self-sustained TB research community in low- and middle-income countries with high TB burdens. (*4*) The Moscow declaration to end TB (2017) and the political declaration of the United Nations high-level meeting on TB (2018) also made bold commitments for action on TB research and innovation. (*3*) In 2020, WHO Member States adopted the Global Strategy for TB Research and Innovation for action to meet these commitments. (*3*)

Intensified TB research, unlike routine TB surveillance and programme activities, is difficult to monitor and evaluate quantitatively. Research varies in type, end-point and outcome, from basic scientific research to operational research. Moreover, research is conducted by the entire scientific community, which includes academia and research institutions that are not necessarily linked to national TB programmes. Bibliometric analysis is widely used in the health sciences and public health (*5*, *6*) to measure scientific productivity and to assess trends and patterns in research output. (*7*, *8*) Bibliometric analyses of research on TB have been reported in several publications, with various objectives. (*9*-*11*) Ramos et al. (*10*) showed increasing research activity in the field of TB during the period 1997–2006 and reported that less research was conducted in countries with the highest estimated numbers of TB cases. Most recently, Nafade et al. (*9*) found that the annual growth rate of TB publications between 2007 and 2016 was 7.3% globally, with the highest rate (13.1%) in Brazil, the Russian Federation, India, China and South Africa (BRICS). No studies are available, however, of regional productivity of research on TB.

The WHO Western Pacific Region (WPR) consists of 37 countries and areas, with a total population of 1.9 billion. The Region is diverse, including only one country with populations of more than 1 billion and small Pacific island countries with a few thousand residents and also countries with high and intermediate TB burdens and others in the pre-elimination stage. The Region accounted for 18% of global TB incidence in 2018. (*1*) The Regional Framework for Action on Implementation of the End TB Strategy in the Western Pacific 2016–2020, (*12*) in line with the End TB Strategy, also emphasized the importance of increasing capacity for research on TB for the development, uptake and optimum use of new interventions and proposed actions such as expanding national TB research networks, developing national TB research plans and priorities, building capacity for TB research and increasing TB research funding.

The aims of this analysis were to: (i) examine regional trends in the numbers of publications on TB indexed in PubMed in the past two decades; (ii) to compare the trends with those for other, selected major infectious diseases; and (iii) to assess intensified TB research activity in countries and areas in the Region quantitatively.

## Methods

A bibliometric analysis was performed with the RISmed package (*13*) in R (CRAN: Comprehensive R Archive Network at https://cran.r-project.org/), which permits extraction of bibliographic content from the United States National Center for Biotechnology Information databases, including PubMed. We extracted metadata from scientific publications indexed in PubMed with a combination of Medical Subject Headings (MeSH) terms for four major infectious diseases, “Tuberculosis,” “HIV Infection,” “Hepatitis” and “Malaria,” and the names of countries and areas in the Western Pacific Region. We then constructed a regional database of the number of publications per year during the period 2000–2019 at 13 September 2020. The four diseases were selected on the basis of the global burden of each as a single infectious disease (*14*) and regional priorities in “reaching the unreached.” (*15*) We did not include countries and areas for which MeSH terms were not available, which were Cook Islands, Kiribati, Marshall Islands, Nauru, Niue, Commonwealth of the Northern Mariana Islands, Solomon Islands, Tokelau, Tuvalu and Wallis and Futuna. The numbers of publications from Pacific island countries and areas were aggregated in the results because of the small number of publications. Duplicates of publications were removed from the regional aggregate counts. In this paper, the numbers of publications from China excluded those from Hong Kong Special Administrative Region SAR (China), Macao SAR (China) and China, Taiwan (China) as they were separately-defined geographical MeSH terms.

We examined trends in the numbers of publications on the four major infectious diseases at regional level over 5-year periods between 2000 and 2019 and computed the percentage increase from the level in 2000–2004, growth rates in each 5-year period and average annual growth rates for the period 2000–2018 (the year 2019 was removed because of the time required to indexing (*16*)). We further examined trends in the numbers of publications on TB in countries over the 5-year periods between 2000 and 2019 and computed the percentage changes from the level in 2000–2004 and average 5-year growth rates. The proportions of publications on TB from China, other regional high-burden countries and non-high-burden countries were also investigated for the same periods.

### Ethics statements

Ethical clearance was not required as this was an analysis of available published research.

## Results

**Figure 1:**
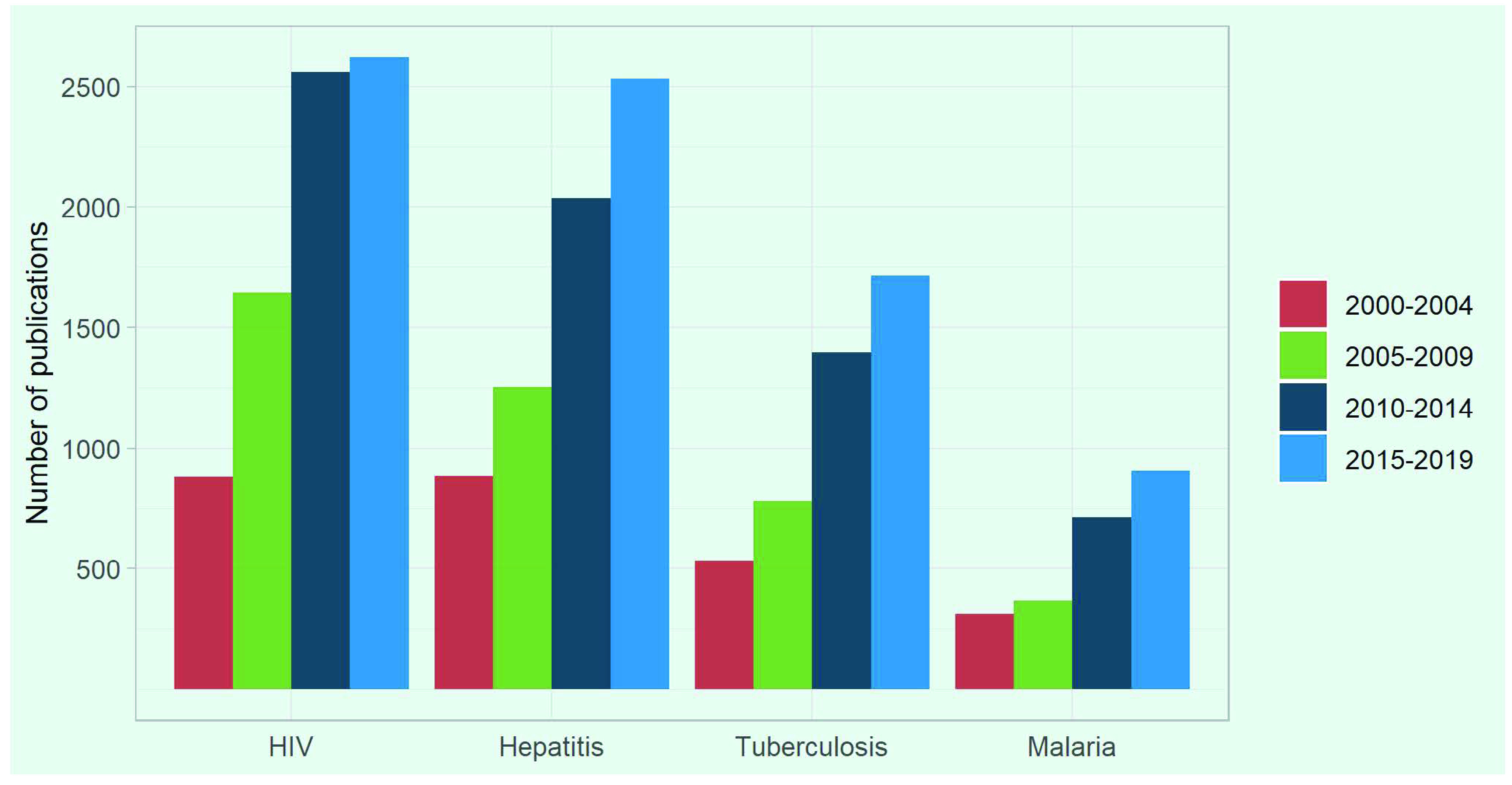
Numbers of publications on major infectious diseases from the WHO Western Pacific Region indexed in PubMed over 5-year periods, 2000–2019

**Figure 2:**
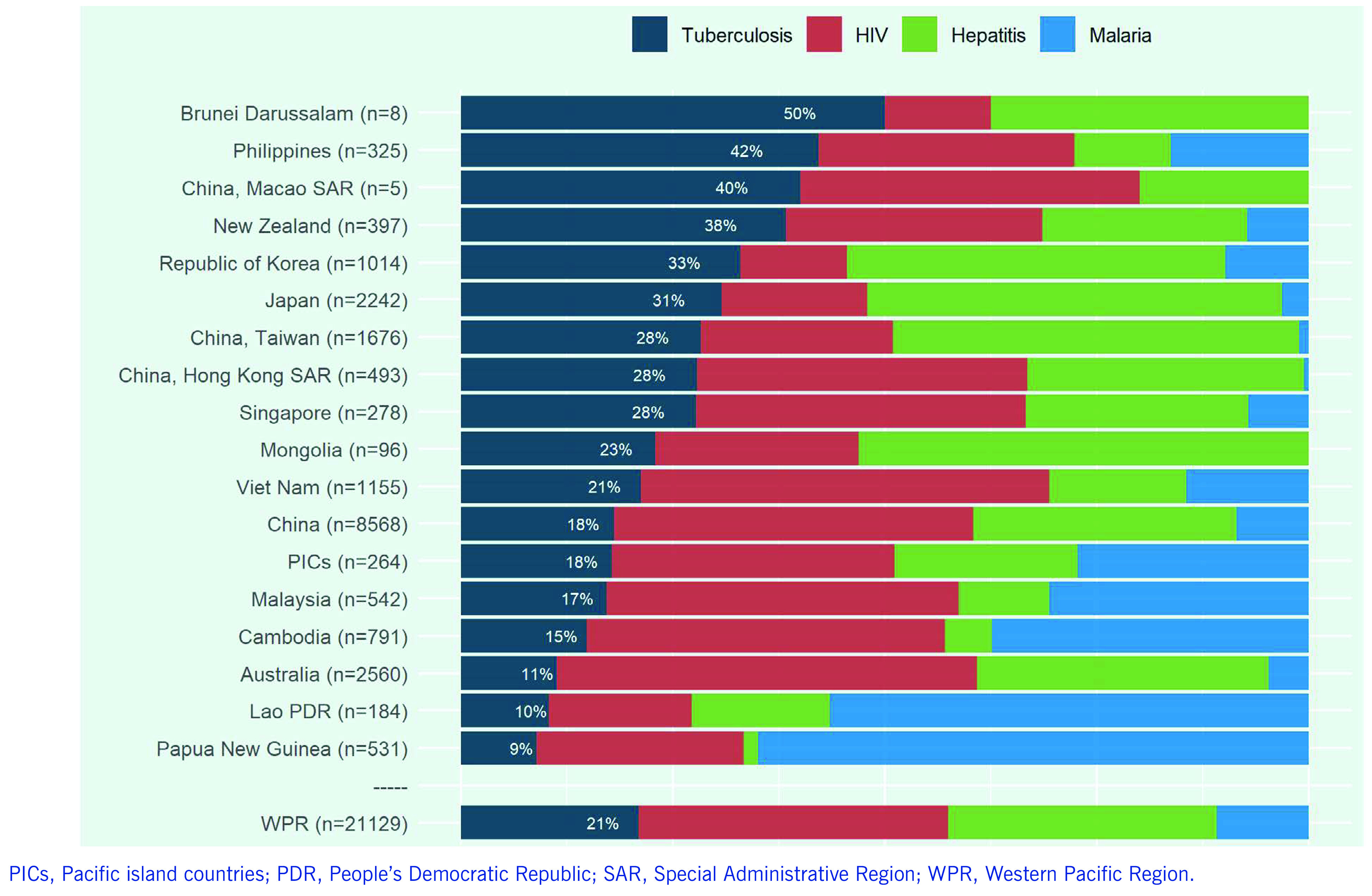
Proportions of publications on TB, HIV, hepatitis and malaria from countries and areas in the Western Pacific Region indexed in PubMed, 2000–2019

The number of publications indexed in PubMed on the four major infectious diseases in countries and areas in the WHO Western Pacific Region has increased by 3.0 times over the past two decades (**Fig. 1**), from 2609 in 2000–2004 to 7770 in 2015–2019 (**Table 1**). During the 5-year periods between 2000 and 2019, articles on HIV were published most often, followed by publications on hepatitis, TB and malaria. In the period 2000–2019, publications on TB accounted for 21% of all publications on the four diseases in the Region, which was less than for HIV (36%) and hepatitis (32%) and more than for malaria (11%) (**Fig. 2**). The proportion of publications on TB varied by country and area, from £10% for Papua New Guinea and the Lao People's Democratic Republic to (*3*)30% for Japan, New Zealand, the Philippines and the Republic of Korea, excluding Brunei Darussalam and Macao SAR (China), which had fewer than 10 publications (**Fig. 2**).

[**insert** Figure 1]

[**insert** Figure 2]

**Table 1:** Numbers and growth rates of publications from the WHO Western Pacific Region indexed in PubMed on major infectious diseases over 5-year periods, 2000–2019

-	Number of publications				% increase, compared to the 2000–2004 level			Growth rate compared to the previous 5-year period			Average annual growth rate for 2000–2018
	2000–2004	2005–2009	2010–2014	2015–2019	2005–2009	2010–2014	2015–2019	2005–2009	2010–2014	2015–2019	
**HIV**	881	1644	2557	2691	187%	290%	297%	87%	56%	2%	10.5%
**Hepatitis**	883	1254	2035	2530	142%	230%	287%	42%	62%	24%	9.1%
**Tuberculosis**	534	781	1396	1714	146%	261%	321%	46%	79%	23%	8.8%
**Malaria**	311	369	714	907	119%	230%	292%	19%	93%	27%	15.6%
**Total**	**2609**	**4048**	**6702**	**7770**	**155%**	**257%**	**298%**	**55%**	**66%**	**16%**	**9.3%**

The number of publications on TB from the Region increased by 3.2 times, from 534 in 2000–2004 to 1714 in 2015–2019 (2.9 times each for HIV, hepatitis and malaria). **Table 1** shows the growth rate in the number of publications on TB in the Region increased by 46% in 2005–2009, 79% in 2010–2014 and 23% in 2015–2019 compared to the previous 5-year period. The average annual growth rate in the number of publications on TB between 2000 and 2018 was 8.8%.

**Figure 3:**
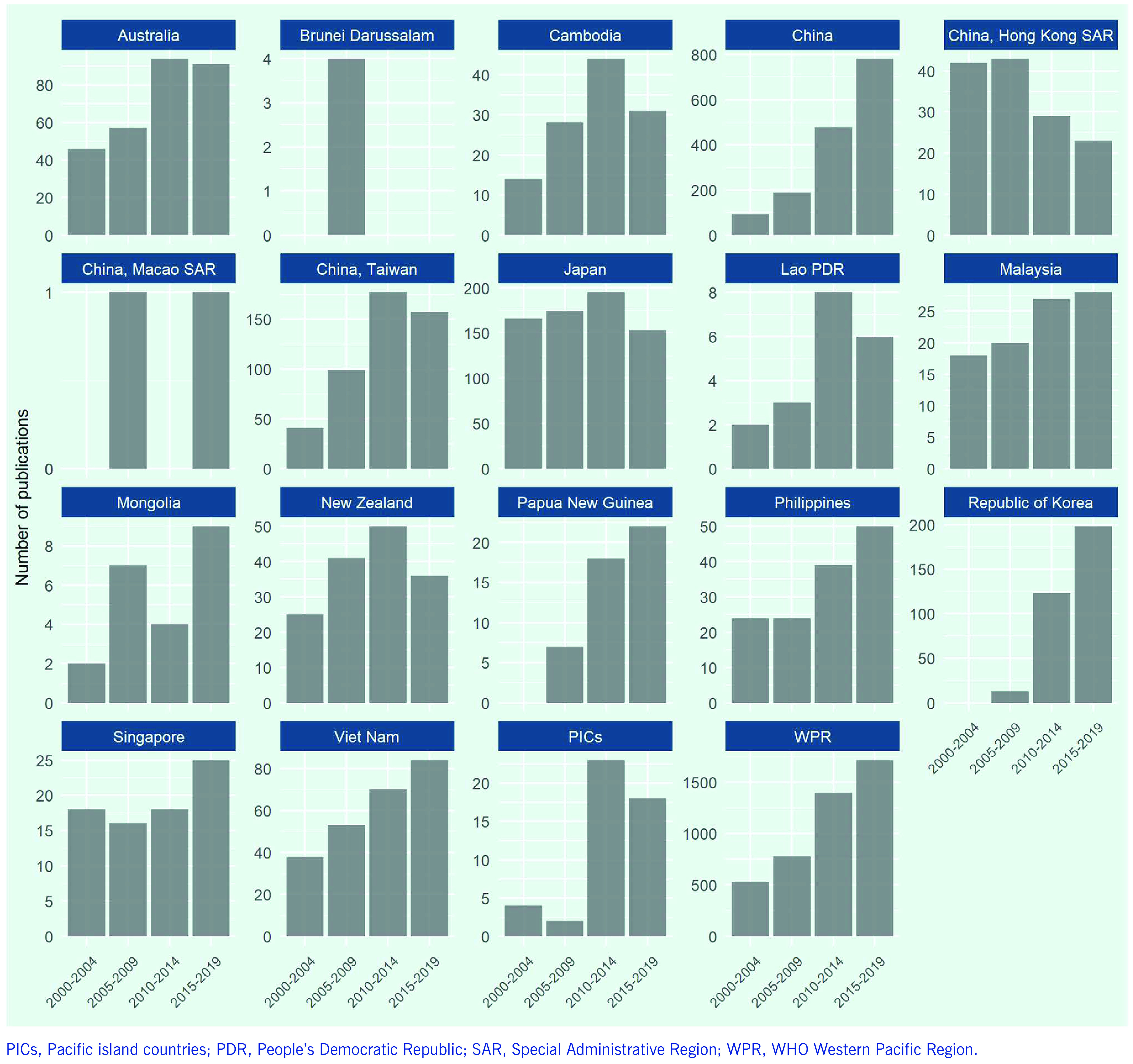
Number of publications on TB from countries and areas in the Western Pacific Region indexed in PubMed over 5-year periods, 2000–2019

**Figure 4:**
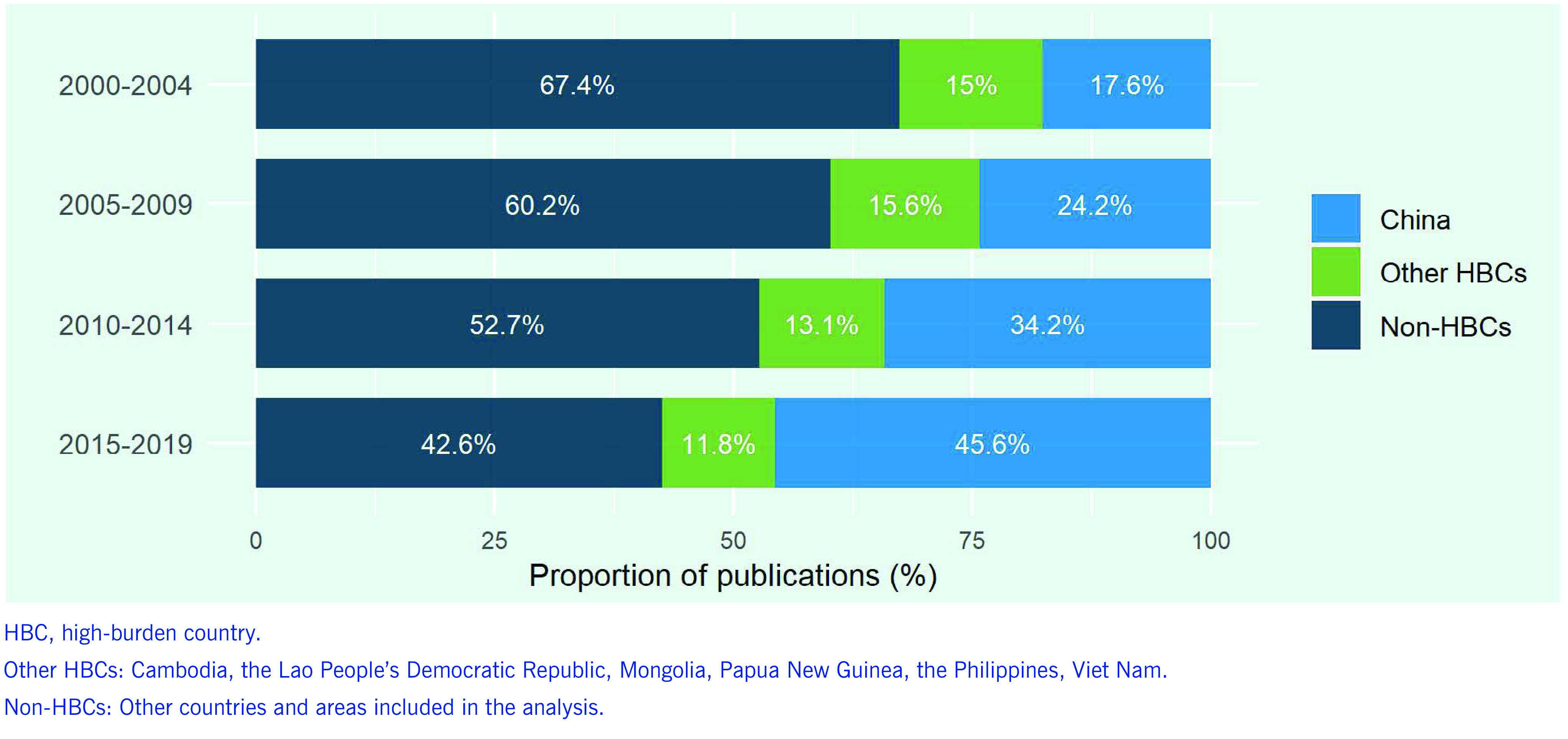
Proportions of publications on TB from China, other high-burden countries and non-high-burden countries/areas in the Western Pacific Region indexed in PubMed over 5-year periods, 2000–2019

Between 2000 and 2019, there were 4425 publications on TB in the Region (**Table 2**). China accounted for the largest proportion (34.8%), followed by Japan (15.5%), China, Taiwan (China) (10.7%), the Republic of Korea (7.5%), Australia (6.5%) and Viet Nam (5.5%). These six countries and areas accounted for > 80% of all publications on TB in the Region; Pacific island countries accounted for only 1%. The number of publications on TB has tended to increase in most countries and areas in the Region in the past two decades, including in Cambodia, Papua New Guinea, the Philippines and Viet Nam, with the highest percentage increase in China (832%)  (**Fig. 3**). The number of publications on TB over the 5-year periods remained at < 10 in the Lao People's Democratic Republic and Mongolia, although increasing trends are observed. The percentage of publications on TB from China out of the total number from the Region increased from 17.6% in 2000–2004 to 45.6% in 2015–2019, while those of other high-TB burden countries and of other countries have shrunk (**Fig. 4**).

[**insert** Figure 3]

[**insert** Figure 4]

**Table 2:** Numbers of publications on TB from countries and areas in the Western Pacific Region indexed in PubMed over 5-year periods, 2000–2019

Country and area	Number of publications					% change from 2000–2004 to 2015–2019	Growth rate between 2010–2014 and 2015–2019
	2000–2004	2005–2009	2010–2014	2015–2019	Total (N/%)		
Australia	46	57	94	91	288 (6.5%)	198%	−3%
Brunei Darussalam	0	4	0	0	4 (0.1%)	N/A	N/A
Cambodia	14	28	44	31	117 (2.6%)	221%	−30%
China	94	189	477	782	1542 (34.8%)	832%	64%
China, Hong Kong Special Administrative Region SAR	42	43	29	23	137 (3.1%)	55%	−21%
China, Macao SAR	0	1	0	1	2 (0.05%)	N/A	N/A
China, Taiwan	41	99	177	157	474 (10.7%)	383%	−11%
Japan	166	174	195	153	688 (15.5%)	92%	−22%
Lao People's Democratic Republic PDR	2	3	8	6	19 (0.4%)	300%	−25%
Malaysia	18	20	27	28	93 (2.1%)	156%	4%
Mongolia	2	7	4	9	22 (0.5%)	450%	125%
New Zealand	25	41	50	36	152 (3.4%)	144%	−28%
Papua New Guinea	0	7	18	22	47 (1.1%)	N/A	22%
Philippines	24	24	39	50	137 (3.1%)	208%	28%
Republic of Korea	0	13	123	198	334 (7.5%)	N/A	61%
Singapore	18	16	18	25	77 (1.7%)	139%	39%
Viet Nam	38	53	70	84	245 (5.5%)	221%	20%
Pacific island countries	4	2	23	18	47 (1.1%)	450%	−22%
**Western Pacific Region**	**534**	**781**	**1396**	**1714**	**4425 (100%)**	**321%**	**23%**

PDR, People’s Democratic Republic; SAR, Special Administrative Region.

N/A = not applicable

## Discussion

Our analysis demonstrates increasing research on the four major infectious diseases, including TB, in the Western Pacific Region over the past two decades. The importance of intensifying research has been stressed in global and regional strategies, not only for TB (*2*, *12*) but also for other communicable disease programmes, including HIV, (*17*) hepatitis (*18*) and malaria. (*19*) It is anticipated that increasing trends in the number of publications reflect accelerated research and innovations to better control and eliminate the diseases.

After 2015, when the End TB Strategy was introduced, the 5-year trend in the number of publications on TB continued to grow, demonstrating successful implementation of the third pillar of the Strategy in the Region. The increasing trends observed in low- and middle-income countries with high burdens of TB, such as Cambodia, China, Papua New Guinea, the Philippines and Viet Nam, may be considered to reflect empowered research communities and enhanced research collaboration on TB in these countries. The annual regional growth rate in the number of publications on TB in 2000–2018 was 8.8%, which was slightly higher than the global annual growth rate of 7.3% for 2006–2017. (*9*)

Government commitment and leadership play pivotal roles in advancing research and innovation for TB, and increasing financial investments is critical. Our analysis showed that China’s contribution to regional TB research productivity was remarkable, especially in 2015–2019. China’s National TB Strategic Plan 2016–2020 emphasizes the importance of intensified national research and development on TB prevention and care and of promoting international cooperation. (*20*) Accordingly, the national annual budget allocated for research and surveys on TB in China increased dramatically between 2015 and 2019, by six times, from  US$ 5.7 million to US$ 34.3 million. (*21*) This may be one reason for the substantial increase in the number of publications, with rapid economic development enhancing domestic research capacity. (*9*, *22*) Ongoing initiatives to intensify collaboration in research on TB within the BRICS TB Research Network (*23*) may accelerate this trend in coming years.

Developing national TB research agendas and strategic plans and establishing national TB research networks creates an environment for high-quality TB research and innovation. (*3*, *12*) In Viet Nam, where the number of publications on TB has increased continuously over the past two decades, the national TB research agenda is explicitly defined in the National TB Strategic Plan 2015–2020. (*24*) Furthermore, in 2015, the Ministry of Health formed the Viet Nam Integrated Centre for TB and Lung Disease Research (VICTORY) under the management of the National Lung Hospital and National Tuberculosis Programme, to lead in implementing and coordinating research on TB and other lung diseases and to establish a research network. (*12*, *25*) This has fostered collaboration on TB research within and outside the country and also led to institutionalized research within programmes to ensure that research outputs inform policy and practice and improve programme performance. (*25*, *26*)

Effective bilateral and multilateral North–South and South–South collaborations among researchers and research institutions in high- and in low- and middle-income countries are essential to promote relevant research and to cross-fertilize research capacity. (*3*) Several TB research networks are active in the WHO Western Pacific Region, including the Centre for Research Excellence in Tuberculosis Control in Australia (*27*) and the Asian Tuberculosis Research and Clinical Trials Integrated Organizational Network among members of the Asia–Pacific Economic Cooperation. (*28*) National TB research institutions, such as the Research Institute of Tuberculosis in Japan and the Korean Institute of Tuberculosis in Republic of Korea, have long contributed to international research on TB and to capacity-building in the Region. Molton et al., (*29*) however, reported less intra-Asian TB research collaboration than in other regions, which they considered a missed opportunity to optimize regional research funding, capacity-building and a region-specific research agenda. Further enhancement of TB research collaboration is desirable in the Region, building on existing networks and initiatives.

Although there is increasing TB research collaboration and productivity in the Region, our analysis indicates that the output remains relatively low in several countries with higher burdens of TB such as Lao People's Democratic Republic and Mongolia as well as in Pacific island countries, where TB incidence per capita can be high. Operational, implementation, health system and social science research on TB to generate context-specific evidence to improve programme performance (*3*) could be prioritized in those countries, coordinated by national programmes, to gain the immediate benefits of research.

Our study has several limitations. First, we included publications only from the PubMed database and only those found with MeSH terms for both diseases and country names. We thus excluded relevant publications in other databases or not indexed as MeSH terms, regional publications from Asia and Oceania with no country-specific indexing and several Pacific island countries for which MeSH terms were not available. Second, the time required for indexing might have affected the completeness of indexing, especially for 2019, although we ensured that sufficient time (257 days) had elapsed between the end date of 2019 and the date of data extraction. These limitations may have resulted in an underestimate of the number of publications. Lastly, we did not investigate regional trends and patterns by research type and programmatic areas in published articles, which could reveal gaps in evidence for the regional TB response. This was beyond the scope of the present analysis but should be addressed in future studies.

Despite these limitations, the results of our bibliometric analysis indicate contemporary trends in TB research productivity in countries and areas in the Region and highlight achievements and gaps in implementing the third pillar of the End TB Strategy. Countries are encouraged to implement the actions proposed in the Global Strategy for TB Research and Innovation to accelerate progress towards ending TB. (*3*) The WHO Regional Office for the Western Pacific will continue to play a catalytic role in fostering regional TB research collaboration and providing technical assistance to build research capacity in national TB programmes in the Region.

## References

[1] Global tuberculosis report 2019. Geneva: World Health Organization; 2019. Available from: https://apps.who.int/iris/bitstream/handle/10665/329368/9789241565714-eng.pdf

[2] Sixty-seventh World Health Assembly. Global strategy and targets for tuberculosis prevention, care and control after 2015. Geneva: World Health Organization; 2014. Available from: https://apps.who.int/gb/ebwha/pdf_files/WHA67-REC1/A67_2014_REC1-en.pdf

[3] Global strategy for tuberculosis research and innovation. Geneva: World Health Organization; 2020. Available from: https://apps.who.int/gb/ebwha/pdf_files/EB146/B146_R7-en.pdf 10.1183/13993003.03539-2020 33184100

[4] A global action framework for TB research in support of the third pillar of WHO’s End TB Strategy. Geneva: World Health Organization; 2015. Available from: https://apps.who.int/iris/bitstream/handle/10665/195772/9789241509756_eng.pdf;jsessionid=CC70274C418150D32845BE57B2E77A74?sequence=1

[5] Clarke A, Gatineau M, Grimaud O, Royer-Devaux S, Wyn-Roberts N, Le Bis I, et al. A bibliometric overview of public health research in Europe. Eur J Public Health. 2007;17 Suppl 1:43–9. 10.1093/eurpub/ckm063 17666422

[6] Sweileh WM, Wickramage K, Pottie K, Hui C, Roberts B, Sawalha AF, et al. Bibliometric analysis of global migration health research in peer-reviewed literature (2000-2016). BMC Public Health. 2018 6 20;18(1):777. 10.1186/s12889-018-5689-x 29925353 PMC6011263

[7] Thompson DF, Walker CK. A descriptive and historical review of bibliometrics with applications to medical sciences. Pharmacotherapy. 2015 6;35(6):551–9. 10.1002/phar.1586 25940769

[8] Xu Z, Yu D. A bibliometrics analysis on big data research (2009–2018). J Data Inf Manag. 2019;1(1-2):3–15. 10.1007/s42488-019-00001-2

[9] Nafade V, Nash M, Huddart S, Pande T, Gebreselassie N, Lienhardt C, et al. A bibliometric analysis of tuberculosis research, 2007-2016. PLoS One. 2018 6 25;13(6):e0199706. 10.1371/journal.pone.0199706 29940004 PMC6016906

[10] Ramos JM, Padilla S, Masiá M, Gutiérrez F. A bibliometric analysis of tuberculosis research indexed in PubMed, 1997-2006. Int J Tuberc Lung Dis. 2008 12;12(12):1461–8. 19017458

[11] Sweileh WM, AbuTaha AS, Sawalha AF, Al-Khalil S, Al-Jabi SW, Zyoud SH. Bibliometric analysis of worldwide publications on multi-, extensively, and totally drug - resistant tuberculosis (2006-2015). Multidiscip Respir Med. 2017 1 11;11(1):45. 10.1186/s40248-016-0081-0 28096979 PMC5225617

[12] Regional framework for action on implementation of the End TB Strategy in the Western Pacific, 2016–2020. Manila: World Health Organization Regional Office for the Western Pacific; 2016. Available from: https://iris.wpro.who.int/bitstream/handle/10665.1/13131/9789290617556_eng.pdf

[13] Kovalchik S. CRAN – package RISmed. [Internet]. Bethesda (MD): National Center for Biotechnology Information; 2017.Available from: Available from https://cran.r-project.org/web/packages/RISmed/RISmed.pdf

[14] James SL, Abate D, Abate KH, Abay SM, Abbafati C, Abbasi N, et al.; GBD 2017 Disease and Injury Incidence and Prevalence Collaborators. Global, regional, and national incidence, prevalence, and years lived with disability for 354 diseases and injuries for 195 countries and territories, 1990-2017: a systematic analysis for the Global Burden of Disease Study 2017. Lancet. 2018 11 10;392(10159):1789–858. 10.1016/S0140-6736(18)32279-7 30496104 PMC6227754

[15] For the future towards the healthiest and safest region. A vision for WHO work with Member States and partners in the Western Pacific. Manila: World Health Organization Regional Office for the Western Pacific; 2020. Available from: https://iris.wpro.who.int/handle/10665.1/14476

[16] Irwin AN, Rackham D. Comparison of the time-to-indexing in PubMed between biomedical journals according to impact factor, discipline, and focus. Res Social Adm Pharm. 2017 Mar-Apr;13(2):389–93. 10.1016/j.sapharm.2016.04.006 27215603

[17] Global health sector strategy on HIV 2016–2021, toward ending AIDS. Geneva: World Health Organization; 2016. Available from: https://www.who.int/hiv/strategy2016-2021/ghss-hiv/en/

[18] Regional action plan for viral hepatitis in the Western Pacific 2016–2020. Manila: World Health Organization Regional Office for the Western Pacific; 2016. Available from: https://iris.wpro.who.int/bitstream/handle/10665.1/13141/97892906177617_eng.pdf

[19] Regional action framework for malaria control and elimination in the Western Pacific (2016–2020). Manila: World Health Organization Regional Office for the Western Pacific; 2017. Available from: https://iris.wpro.who.int/handle/10665.1/13578

[20] 13th five-year national TB prevention and treatment plan. Beijing: General Office of the State Council of the People’s Republic of China; 2017.

[21] Global tuberculosis database [Internet]. Geneva: World Health Organization; 2020. Available from: https://www.who.int/tb/country/data/download/en/

[22] Bai J, Li W, Huang YM, Guo Y. Bibliometric study of research and development for neglected diseases in the BRICS. Infect Dis Poverty. 2016 9 6;5(1):89. 10.1186/s40249-016-0182-1 27595987 PMC5011792

[23] Castor K, Mota FB, da Silva RM, Cabral BP, Maciel EL, de Almeida IN, et al. Mapping the tuberculosis scientific landscape among BRICS countries: a bibliometric and network analysis. Mem Inst Oswaldo Cruz. 2020 3 16;115(1):e190342. 10.1590/0074-02760190342 32187325 PMC7066990

[24] National strategic plan for tuberculosis control for the period 2015–2020. Hanoi: National Tuberculosis Control Programme Viet Nam; 2014.

[25] Hoa NB, Nhung NV, Kumar AMV, Harries AD. The effects of placing an operational research fellow within the Viet Nam National Tuberculosis Programme. Public Health Action. 2016 12 21;6(4):273–6. 10.5588/pha.16.0044 28123967 PMC5176054

[26] Hoa NB, Nhung NV. National tuberculosis patients cost survey: research findings lead to change in policy and practice, Viet Nam. Public Health Action. 2019 6 21;9(2):50–2. 10.5588/pha.18.0082 31417852 PMC6645445

[27] Centre of Research Excellence in Tuberculosis Control. Available from: https://www.tbcre.org.au/, accessed 7 September 2020.

[28] The APEC health working group’s work plan for 2019. Asia–Pacific Economic Cooperation; 2019. Available from: https://www.google.com/url?sa=t&rct=j&q=&esrc=s&source=web&cd=&ved=2ahUKEwiQo7Oz_dXrAhVQQd4KHb9wBJwQFjABegQIARAB&url=http%3A%2F%2Fapec.org%2F-%2Fmedia%2FFiles%2FGroups%2FHRD%2FEndorsed-2019-HWG-Work-Plan.docx&usg=AOvVaw2lzQdymKv-A17vSVIc6uLP

[29] Molton JS, Singh S, Chen LJ, Paton NI. International tuberculosis research collaborations within Asia. BMC Res Notes. 2017 9 7;10(1):462. 10.1186/s13104-017-2769-4 28882158 PMC5590202

